# Monitoring of Airborne Mercury: Comparison of Different Techniques in the Monte Amiata District, Southern Tuscany, Italy

**DOI:** 10.3390/ijerph17072353

**Published:** 2020-03-31

**Authors:** Valentina Rimondi, Renato Benesperi, Marc W. Beutel, Laura Chiarantini, Pilario Costagliola, Pierfranco Lattanzi, Daniela Medas, Guia Morelli

**Affiliations:** 1 Dipartimento di Scienze della Terra, Università di Firenze, Via G. La Pira 4, 50121 Firenze, Italy; laura.chiarantini@unifi.it (L.C.); pilario.costagliola@unifi.it (P.C.); 2 CNR-IGG, Via G. La Pira 4, 50121 Firenze, Italy; pierfrancolattanzi@gmail.com (P.L.); guia.morelli@igg.cnr.it (G.M.); 3 Dipartimento di Biologia, Università di Firenze, Via G. La Pira 4, 50121 Firenze, Italy; renato.benesperi@unifi.it; 4 University of California, Merced, 5200 Lake Road, Merced, CA 95343, USA; mbeutel@ucmerced.edu; 5 Centro di Servizi di Microscopia Elettronica e Microanalisi (M.E.M.A), Università di Firenze, Via G. Capponi 3r, 50121 Firenze, Italy; 6 Dipartimento di Scienze Chimiche e Geologiche, Università di Cagliari, S.S. 554 bivio per Sestu, 09042 Monserrato (CA), Italy; dmedas@unica.it

**Keywords:** biomonitoring, airborne pollutants, particulate Hg, lichens, tree barks, passive air samplers, mining area

## Abstract

In the present study, mercury (Hg) concentrations were investigated in lichens (*Flavoparmelia caperata* (L.) Hale, *Parmelia saxatilis* (L.) Ach., and *Xanthoria parietina* (L.) Th.Fr.) collected in the surrounding of the dismissed Abbadia San Salvatore Hg mine (Monte Amiata district, Italy). Results were integrated with Hg concentrations in tree barks and literature data of gaseous Hg levels determined by passive air samplers (PASs) in the same area. The ultimate goal was to compare results obtained by the three monitoring techniques to evaluate potential mismatches. Lichens displayed 180–3600 ng/g Hg, and Hg concentrations decreased exponentially with distance from the mine. Mercury concentration was lower than in *Pinus nigra* barks at the same site. There was a moderate correlation between Hg in lichen and Hg in bark, suggesting similar mechanisms of Hg uptake and residence times. However, correlation with published gaseous Hg concentrations (PASs) was moderate at best (Kendall Tau = 0.4–0.5, *p* > 0.05). The differences occurred because a) PASs collected gaseous Hg, whereas lichens and barks also picked up particulate Hg, and b) lichens and bark had a dynamic exchange with the atmosphere. Lichen, bark, and PAS outline different and complementary aspects of airborne Hg content and efficient monitoring programs in contaminated areas would benefit from the integration of data from different techniques.

## 1. Introduction

Mercury (Hg) is a widespread contaminant of much concern due to its high toxicity, persistence, and accumulating behavior in the environment [1]. In the reduced form (Hg^0^), Hg displays long residence times in the atmosphere and a consequent ability to be transported over long distances, making it a global scale pollutant [2]. Now more than ever, the coming into force of the Minamata convention requires the proper monitoring of airborne Hg contents in order to reduce Hg anthropogenic emissions. An effective estimation of environmental and human health risks to Hg exposure depends on the development of reliable, low cost, easy-to-use monitoring networks [3]. 

Traditionally, the monitoring of atmospheric Hg has been accomplished by instrumental techniques, like Light Detection and Ranging (LIDAR; [4]), and compact analyzers, such as Tekran® and Lumex® instruments [5]. These methods are excellent to characterize point sources [6]. However, they lack spatial resolution and provide only short-term information on Hg contamination [6,7].

Biomonitoring is the assessment of gradient of pollutants in naturally occurring living biological material [8]. Among wildlife, mammals, birds, fish, and most recently snakes have been employed as biomarkers for Hg [9]. In plants, Hg especially accumulates in lichens, algae and mosses, and in higher plants. The employment of Hg concentrations in lichens and mosses in situ or after transplantation is a long-established practice for Hg monitoring [10,11,12,13,14,15]. Recently, the use of higher plant tissues (e.g., tree rings, bark, leaves) has also increased [13,16,17]. In addition to the determination of total Hg in plant tissues, some studies also investigate the adverse effects (such as alteration in transpiration and photosynthesis, imbalance in carbohydrate metabolism and production of secondary stresses) on the growth and metabolism of plants [18].

A recently developed technique for Hg monitoring is represented by manufactured passive air samplers (PASs), where a bituminous coal-derived, sulfur-impregnated, activated carbon is employed as sorbent for Hg species [19]. Passive sampling, either by employing living organisms or manmade products, is a more sustainable and ecologically relevant approach to monitor Hg for longer periods of time [20,21,22] and allows a better detection of nonpoint sources of Hg and better spatial resolution [6,7]. 

The different monitoring techniques, relying on different processes, do not necessarily give compatible results. Hence, several attempts have been made to evaluate the reliability and comparability of technical and biological methods to monitor Hg in the atmosphere [8], as well as the interchangeability of substrata. For example, epiphytic lichens in mining areas were found to accumulate more Hg than mosses due to differences in morphology and ecophysiology [23,24]. This has led some authors to conclude that the best monitoring programs are achieved using more than one substratum [25]. To date, studies comparing Hg contents in tree barks and epiphytic lichens collected at the same sites are not abundant [26,27,28,29] and have been mostly conducted in areas with low concentrations of Hg in air. Additionally, no studies have compared lichens and tree barks with the new recently developed PAS system. To fill this gap, epiphytic lichens and tree barks (*Pinus nigra* J.F. Arnold) were investigated for Hg concentrations in the Monte Amiata area (Tuscany, Italy), a regional (possibly global) scale hotspot for Hg because of the presence of a large abandoned mining and smelting district. These data were compared with the gaseous Hg concentrations obtained by PASs on the same area. The comparison was based on a limited dataset and should be regarded as preliminary. However, it sets the ground for an optimal, and possibly complementary, use of the different techniques in monitoring programs.

## 2. Materials and Methods

Monte Amiata (42°53′00’’N 11°37′00’’E) is an extinct Quaternary (ca. 300 Ka) volcano reaching an elevation of 1738 m a.s.l. The surrounding area hosts the third largest Hg mining and production district worldwide (>100,000 tons metal produced), and a present day geothermal field [30]. Mercury mining and smelting operated in the area from 1848 to 1982, although the main production occurred from 1900 to 1970 [31]. The largest mine and smelting plants were near the town of Abbadia San Salvatore (ASSM), located on the eastern slope of the volcano. The environmental legacy of this extensive Hg mining and metallurgy has been the subject of many studies (see [30] for a partial reference list).

**Figure 1:**
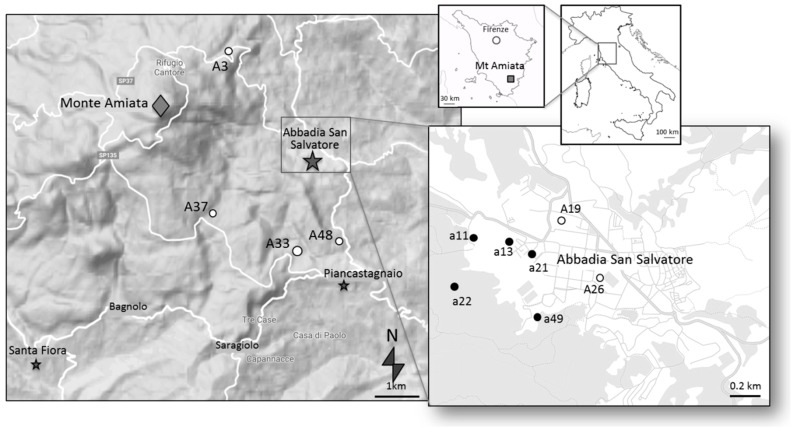
(**a**) Location of sampling sites in the Monte Amiata area; (**b**) zoom of sampling sites in the Abbadia San Salvatore town.

Lichen sampling was performed concurrently with tree bark sampling at 10 sites (Figure 1) in July 2016, making reference to grids established for deployment of PASs [6]: a closer-spaced grid near ASSM (Abbadia grid; sites identified by lowercase a and a progressive number, e.g., a1), and a larger, wider-spaced grid (Amiata grid; sites identified by capital A and a number, e.g., A1), covering an area of 41.6 km^2^. Having previously decided to sample *Pinus nigra* trees (to be consistent with the previous study of [16]), the site choice was conditioned by the presence of this species, making efforts to select trees as close as possible to a PAS deployment site. We obtained samples of *Flavoparmelia caperata* (L.) Hale, *Parmelia saxatilis* (L.) Ach., and *Xanthoria parietina* (L.) Th.Fr., which were taken whenever possible from the same tree sampled for barks by previous research [32], or at least from a nearby location, at about the same height (approximately 100 cm to 150 cm). It was only possible to collect more than one lichen species at some sites. We estimated that at each site, the lichen samples, the tree sampled for bark, and the next PAS were all within a maximum 20 m distance (usually less). Collected lichen samples were identified in the field and subsequently verified in the laboratory using a dissecting microscope. The nomenclature of lichens followed [33]. For Hg concentrations, we decided to use the whole lichen thallus of each sample in order to compare the long-term accumulation performance of the two substrates (lichens and barks).

Lichen thalli were airdried and carefully cleaned under a dissecting microscope. Then, the lichen material was manually shredded into small pieces and homogenized using a ceramic mortar to obtain three replicates of about 40 mg for each collected species in each site. Lichens were analyzed for total Hg in triplicate on a Milestone DMA-80 (USEPA method 7473). Results were then expressed as the mean *±* standard deviation. The local gaseous elemental Hg concentrations (GEM) in the air were taken from [6]. A description of the analytical and computation methods and a description of PAS have been provided by the authors of [6] and [19], respectively. As reported by the authors of [6], deployment times were different for the Amiata and the Abbadia grids. We did not know the specific value of GEM at the moment of bark and lichen sampling. However, the range of values in the time interval including our sampling was fairly narrow. Therefore, they can be assumed to be representative of conditions at sampling time, at least as an order of magnitude (see further discussion).

Because of the small number of samples, linear correlation between variables was explored by the use of the nonparametric Kendall’s Tau test.

## 3. Results

Table 1 summarizes, for each site, the Hg contents (dry weight) in soil, lichens, and Pinus nigra barks, and the range of elemental Hg concentrations in air estimated from PAS data. For the reasons explained by the authors of [32], for barks, the Hg concentration was the mean of the four samples taken at 150 cm above ground. Mercury concentrations in lichens (180–3,600 ng/g) were, in general, of the same order of magnitude for the three recognized species (*Flavoparmelia caperata* (L.) Hale, *Parmelia saxatilis* (L.) Ach., and *Xanthoria parietina* (L.) Th.Fr.). For the three sites (a13, a21, and a49), where more than one lichen species was collected, we did not observe a consistent order of enrichment by species. For example, at each of the three sites, a different species exhibited the highest Hg content (*X. parietina*, *P. saxatilis*, and *F. caperata*, respectively). Mercury contents of both *X. parietina* and *F. caperata* showed a good (Tau = 0.8), statistically significant (*p* = 0.05) correlation with those of the corresponding soil. For *P. saxatilis*, which had only four sample pairs, we did not perform the calculation.

**Figure 2:**
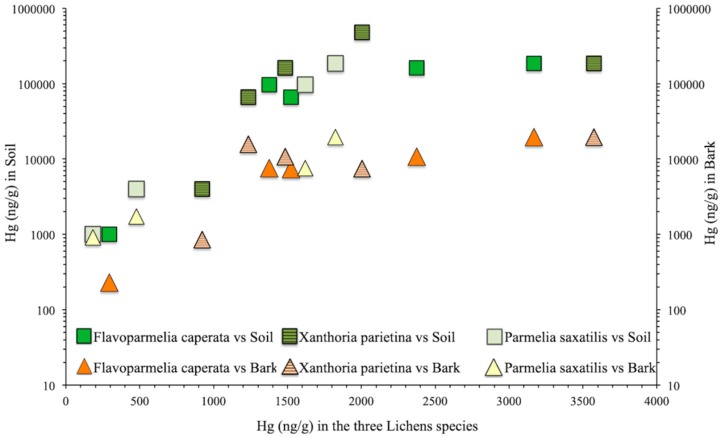
Correlation between Hg contents in lichens, barks, and soils.

Lichen concentrations were consistently lower (up to an order of magnitude) than bark concentrations (850–19,500 ng/g) at the same location, with the exception of site A48, where concentrations were very similar (230 and 290 ng/g for barks and lichens, respectively). In a bivariate plot (Figure 2), Hg concentrations in lichens and barks appear positively correlated. The correlation was strong (Tau = 0.8) and statistically significant (*p* = 0.05) for the species *F. caperata*, while the correlation was weak (Tau = 0.4) and not significant (*p* = 0.3) for *X. parietina*.

For gaseous Hg concentrations, the highest values were observed at site a49, which was located close to the mine (Figure 1b). Consistently, lower values (<5 ng/m^3^) were found in the Amiata grid (Figure 1a), located at background sites with respect to the pollution source (Abbadia Hg mine). However, the correlation between Hg in bark/Hg in lichens and gaseous Hg was moderate (Tau = 0.4–0.5, *p* > 0.05).

## 4. Discussion

**Figure 3:**
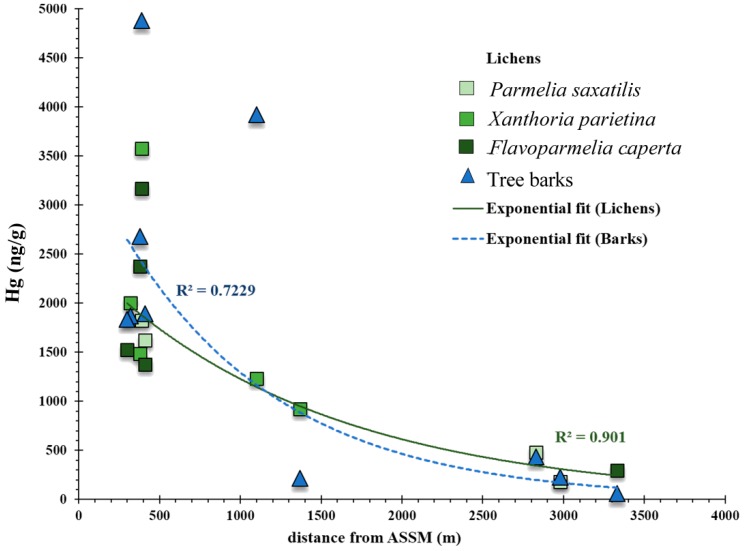
Variations of Hg contents in barks and lichens as a function of distance from Abbadia San Salvatore (ASSM) (consistent with [6], the origin was taken at their site a32). For a better graphical comparison, the values for barks were divided by four. At sites where more than one lichen species was present, the fit was calculated considering the average of values.

Mercury concentrations in lichens were the same order of magnitude as those previously reported for lichen species in the Monte Amiata area [34,35,36,37]. Specifically, for *X. parietina*, the concentrations reported here (920–3,600 ng/g) were mostly higher than those (10–1,960 ng/g) reported by the authors of [37]. Notably, these authors analyzed only the outermost part of the lichens. On the other hand, very high concentrations (up to 40 mg/kg) were reported by the authors of [34,35] in samples of *Parmelia sulcata* directly exposed to ventilations shafts of ASSM. Those authors also showed a decrease of Hg contents lichen with distance from ASSM similar to our data (compare Figure 3 in [34] with our Figure 3). Compared to other worldwide mining locations, Hg contents in lichens from Monte Amiata were slightly lower than the values (up to 4,500 ng/g Hg) found in native lichens sampled in the Almadén Hg district in Spain [38], but generally higher than the range of 60–520 ng/g reported for Nova Scotia, where gold mining was active from 1861 to 1942 [7]. 

Mercury content in lichen showed a good correlation with local Hg soil content, which is consistent with previous reports [35]. Working on a slightly larger bark sample set, a statistically significant correlation was established between Hg contents in barks and soil [32]. The comparison of lichen and bark data showed that the Hg scavenging efficiency per mass unit was generally higher for barks. By contrast, in other studies (dealing with different lichen and plant species), Hg contents were found to be higher in lichens than in tree barks [26,27,28,29]. At present, the mechanism regulating Hg uptake by lichens or bark is unknown. However, the fairly good correlation between the contents in the two matrices suggests that uptake mechanisms may be largely similar. Both substrates may take up Hg as gaseous species (mostly Hg^0^) and as particulate matter. As reviewed by the authors of [39], Hg^0^ taken up by lichens could be rapidly oxidized to water-soluble Hg^2+^ by catalase, an enzyme involved in cell protection against oxidative stress, and Hg contained in adsorbed particulate matter may also be transformed by organic radicals into different compounds. There is no comparable knowledge of transformations occurring in Hg speciation in bark, but preliminary results have showed a certain degree of Hg binding with organic functional groups (mainly thiols) [40]. Both lichens and barks show a dynamic exchange with the atmosphere, i.e., they can release part of the Hg taken up. This release can occur through (a) mechanical removal by wind/rain of previously adsorbed particles (e.g., [41]), (b) dissolution by rain of soluble species, and (c) re-emission of gaseous Hg [42,43], a phenomenon which is enhanced in summer. In general, however, the released Hg is only a small fraction of that taken up. For instance, the authors of [32] observed a negligible release of Hg by *Pinus nigra* bark upon 24 h of batch reaction with deionized water. The residence time of the metal in lichen was estimated to be in the order of a few years (references in [39]). For bark, there are no data, but a weak correlation between Hg bark content and tree age (tens of years) was observed [32], suggesting that the residence time may be significantly shorter than tree age (i.e., possibly similar to lichen).

By contrast, PASs rapidly and irreversibly trap only gaseous Hg compounds. The upper limit of the uptake capacity of the carbon sorbent is very high, and it was certainly not exceeded during the deployment at ASSM [6]. The differences between PAS-derived gaseous concentrations and lichen/bark Hg contents may therefore be ascribed to the following factors: (a) lichens and barks picked up Hg-bearing solid particles. This contribution is presumably more important near contaminated soils (at Monte Amiata, soils contained ore minerals and/or smelting products, for instance, the presence of β-HgS in barks was demonstrated by [40]); (b) lichens and barks dynamically exchanged Hg with the atmosphere. This exchange makes lichen not very reliable indicators of Hg concentrations in air [39]; (c) some of the concentrations obtained with PAS referred to time period of one week only, and the specific wind and temperature conditions prevailing during one of the week-long sampling periods (July 2016) may not be reflective of the long-term average conditions [6]; (d) the time period reflected by the different types of samples was not the same. Whereas PAS measurements represented conditions for clearly defined deployment periods (of one week or three months in the current case), the time period represented by the biological samples was less clearly defined. This can cause discrepancies if there are long-term trends in the concentrations; (e) as previously noted, PAS and sampled lichens/barks were always fairly close, but never exactly coincident. However, we believe that this last factor was of negligible impact. Indeed, PAS data [6] suggest that the spatial air concentration variability is rather smooth and gradual rather than noisy and spotty, so we would not expect very large differences in concentrations over a small scale. Considering these factors, differences in the overall trends, depicted by all three indicators, should not be interpreted as inconsistencies. It is possible that they are all reliable witnesses of different aspects of airborne Hg contamination. PAS give more accurate estimates of short-term local gaseous Hg concentrations, whereas lichens and barks give a measure of long-term Hg pollution near ground level, including deposition of airborne particulate. 

Gaseous Hg is the predominant (> 99%) form of Hg from natural emissions [44]. However, in mine locations like that of Abbadia, particulate Hg may be significant due the presence of mine waste next to the town center, where particulate Hg can be rapidly dispersed by local winds to the surroundings. The direct measurement of particulate Hg is an expensive task, requiring sophisticate instruments such as Tekran. The employment of barks and/or lichens can offer preliminary, spatially extended information on the presence of such species at a low cost. Once the presence of particulate Hg is indicated, more detailed specific investigations may be set up for this purpose. 

## 5. Conclusions

Comparison of Hg contents in epiphytic lichens and *Pinus nigra* barks with gaseous Hg concentrations in air calculated from PAS data at the same locations in the Monte Amiata area led to the following conclusions: barks accumulated higher Hg per unit mass with respect to lichens of the same area. However, there was an overall good agreement between lichen and bark data, suggesting similar mechanisms of Hg uptake and residence times a significant fraction of Hg in bark and lichens was likely present in the particulate form due to soil resuspension. PAS-derived gaseous concentrations showed a moderate match with lichen/bark concentrations. The main factors contributing to differences included a partly different speciation of trapped Hg, the dynamic exchange of Hg with the atmosphere, and the rapid response of PAS to local variations compared to longer residence times in lichen and bark the overall consistency of Hg distribution trends depicted by the three methods suggests that they can be usefully integrated in monitoring programs. We suggest that barks or lichens may be successfully applied to provide preliminary indirect information on the presence of particulate Hg in different case studies (mining and urban areas, for example) before the employment of more sophisticated and expensive techniques which can lead to its quantification.

## Figures and Tables

**Table 1:** Data of Hg contents in lichens (this study) in tree barks, soils, and Hg° in air (literature data).

Site	Hg in lichens (ng/g)			Hg in bark ^1^ (ng/g)	Hg in soil ^1^ (mg/kg)	Hg in air (ng/m^3^) ^2^
	*Parmelia saxatilis*	*Xanthoria* *parietina*	*Flavoparmelia caperata*			Range
A3	480 *±* 110	-	-	1700 *±* 400	3.7 *±* 0.1	1.68–1.84
A19	-	1200 *±* 280	-	15,700 *±* 4200	66 *±* 0.4	3.82–4.24
A33	-	920 *±* 300	-	850 *±* 280	4.1 *±* 0.4	2.67–3.26
A37	180 *±* 50	-	-	920 *±* 330	1.1 *±* 0.1	1.86–2.07
A48	-	-	290 *±* 50	230 *±* 110	1.5 *±* 0.1	2.03–3.03
a11	-	2000 *±* 450	-	7500 *±* 980	480 *±* 1	9.86–15.7
a13	1800 *±* 150	3600 *±* 820	3200 *±* 430	19,500 *±* 2700	186 *±* 1	16–17.9
a21	1600 *±* 300	-	1400 *±* 230	7600 *±* 500	97 *±* 1	11.6–17.8
a22	-	-	1500 *±* 130	7300 *±* 1300	66 *±* 3	7.48–14.8
a49	-	1500 *±* 120	2400 *±* 330	10,700 *±* 700	163 *±* 1	24.7–116

^1^ from [32]; ^2^ data for June 30 through July 6, 2016 (a11–a49) and from July 4 through October 10, 2016 (A3–A48) from [6].
